# Atlantic salmon (*Salmo salar* L.) genetics in the 21st century: taking leaps forward in aquaculture and biological understanding

**DOI:** 10.1111/age.12748

**Published:** 2018-11-14

**Authors:** R. D. Houston, D. J. Macqueen

**Affiliations:** ^1^ The Roslin Institute and Royal (Dick) School of Veterinary Studies The University of Edinburgh Midlothian EH25 9RG UK; ^2^ School of Biological Sciences University of Aberdeen Aberdeen AB24 2TZ UK

**Keywords:** evolutionary genetics, genome editing, genomics, salmonid, selective breeding, sequencing technology, whole genome duplication

## Abstract

Atlantic salmon (*Salmo salar* L.) is among the most iconic and economically important fish species and was the first member of Salmonidae to have a high‐quality reference genome assembly published. Advances in genomics have become increasingly central to the genetic improvement of farmed Atlantic salmon as well as conservation of wild salmon stocks. The salmon genome has also been pivotal in shaping our understanding of the evolutionary and functional consequences arising from an ancestral whole‐genome duplication event characterising all Salmonidae members. Here, we provide a review of the current status of Atlantic salmon genetics and genomics, focussed on progress made from genome‐wide research aimed at improving aquaculture production and enhancing understanding of salmonid ecology, physiology and evolution. We present our views on the future direction of salmon genomics, including the role of emerging technologies (e.g. genome editing) in elucidating genetic features that underpin functional variation in traits of commercial and evolutionary importance.

## Introduction

Atlantic salmon (*Salmo salar* L.) (hereafter ‘salmon') is among the most famous and economically important fish species globally. In addition to being a prized sport fish with a fascinating life cycle, major ecological importance and high conservation value, salmon is a nutritious food product farmed for human consumption. Salmon aquaculture is worth approximately 8.5 billion GBP (~9.7 billion Euro) annually (FAO 2017) and contributes significantly to food, economic and employment security in many nations, especially Norway, Chile, Canada and the United Kingdom. Genetics and genomics have key roles in the current and future management of farmed and wild salmon stocks. Consequently, huge research investment, often supported by industry, is driving the field forward at a remarkable pace. Fuelled by the recent publication of a high‐quality reference genome for salmon (Lien *et al*. 2016) and related species from the Salmonidae family (e.g. Christensen *et al*. 2018a,2018b; Narum *et al*. 2018), there is currently a major interest in applying genome‐wide tools to enhance selective breeding for aquaculture and improve knowledge of genome biology, physiology, ecology and evolution (Macqueen *et al*. 2017). The goal of this article is to provide an overview of Atlantic salmon and its key genetic features before reviewing the current and future research landscape in genetics and genomics.

## Evolutionary history and key genetic features

### Phylogeny and macroevolution

Atlantic salmon is one of two recognized *Salmo* species, the other being brown trout (*Salmo trutta*). *Salmo* sits within the Salmoninae subfamily, which also includes *Oncorhynchus* (Pacific salmons), *Salvelinus* (charrs), *Parahucho* (Sakhalin taimen), *Hucho* (huchens/taimens) and *Brachymystax* (lenoks). The position of *Salmo* within Salmoninae has been long‐debated, but a recent study used genome‐wide markers to affiliate *Salmo* and *Parahucho* as a sister group (Lecaudey *et al*. 2018). Evidently, *Salmo* and *Parahucho* split approximately 22 million years ago, whereas their ancestor diverged from a group containing *Oncorhynchus* and *Salvelinus* approximately 10 million years earlier (Lecaudey *et al*. 2018). The clade that includes *Salmo*,* Parahucho*,* Oncorhynchus* and *Salvelinus* shares a capacity for anadromy (Alexandrou *et al*. 2013)—the ability to migrate into seawater after spending early life in streams and rivers. This trait likely evolved after divergence from the *Hucho*‐*Brachymystax* lineage, for which the full life‐cycle is spent within freshwater, a feature present in more distantly related lineages including graylings (*Thymallinae*), along with *Eosalmo* (extinct), the earliest known salmonid in the fossil record (Wilson & Li 1999). Consequently, the famous ability of salmon to transform their juvenile physiology, migrate to and exploit oceanic feeding grounds—sometimes thousands of miles from their birthplace—has ancient evolutionary origins. This life‐history strategy was also proposed to have driven species diversification (Macqueen & Johnston 2014), and its evolution may be linked to genetic features distinguishing salmonids from other fishes (see the later sub‐section ‘The “Ss4R” WGD event').

### Intra‐specific diversity and microevolution

Salmon have a broad distribution in the Northern hemisphere and diverged into North American and European lineages at least 0.6–0.7 million years ago (King *et al*. 2007), with an even deeper divergence 1.56—1.76 million years ago suggested recently (Rougemont & Bernatchez 2018). These lineages are characterized by notable differences in chromosomal organization (Hartley 1987) and mating incompatibilities (Cauwelier *et al*. 2012), so they can be reasonably classified as sub‐species (King *et al*. 2007; Rougemont & Bernatchez 2018). Substantial structure exists within each lineage, including three differentiated European clades and several North American groups (Bourret *et al*. 2013; Moore *et al*. 2014). There is evidence of substantial recent gene flow between and within these major lineages and sub‐populations (Rougemont & Bernatchez 2018).

Salmon encounter diverse environments across their range. Coupled with a strong tendency to reproduce in the streams of their birth, populations show significant genetic differentiation and adaptation at small spatial scales (Garcia de Leaniz *et al*. 2007; Fraser *et al*. 2011). Although most salmon maintain anadromous life‐history strategies, populations on both continents have become trapped in post‐glacial freshwater systems. These ‘landlocked' fish have experienced rapid genetic and phenotypic differentiation owing to drift coupled with selection on a distinct set of traits, for example a loss/reduction in selection on the systems that prepare anadromous populations for seawater entry (e.g. Nilsen *et al*. 2008). Although rapid phenotypic change and plasticity in response to new environments is a highly recognized feature of many salmonids (e.g. Klemetsen *et al*. 2003), the mechanisms involved remain poorly understood. However, genomic plasticity provided by an ancestral whole‐genome duplication (WGD) event (see the next subsection) has been linked to the salmon's high capacity for adaptation (Kjærner‐Semb *et al*. 2016).

### The ‘Ss4R' WGD event

The salmon's nuclear material sums to approximately 3 billion bases and contains 50–60% repeat content (Lien *et al*. 2016). Although this closely matches the human genome, the basis for these features differs markedly. The ancestor to salmonids experienced an autotetraploidization WGD (Allendorf & Thorgaard 1984) 88–103 million years ago (Macqueen & Johnston 2014), an event coined ‘Ss4R' (salmonid‐specific 4th round of WGD) to account for additional rounds of WGD at the base of teleost fishes (‘teleost‐specific' Ts3R) (Glasauer & Neuhauss 2014) and the vertebrate lineage (1R/2R) (Dehal & Boore 2005).

Autotetraploidization involves spontaneous doubling of all chromosomes, distinct from the other major WGD class, allotetraploidization, which involves hybridisation of distinct species. After the latter, the two genomes within a cell are usually different enough to segregate into two sets of bivalents during meiosis, which rescues pairing incompatibilities among hybridizing species prior to WGD (Otto 2007). Conversely, autotetraploidization leads to four chromosome sets that initially pair randomly during meiosis after WGD; preferential bivalent pairing must be re‐established before duplicated genes created by WGD can diverge beyond an allelic state (Martin & Holland 2014; Lien *et al*. 2016; Robertson *et al*. 2017). This represents one of the key outcomes of rediploidization, the process whereby a tetraploid genome returns to diploidy. The re‐establishment of bivalent pairing in salmonids involved large structural reorganizations (e.g. inversions) associated with bursts of transposable element proliferation, suggesting that Ss4R resulted in relaxed ‘policing' of deleterious transposable element propagation (Lien *et al*. 2016). Remarkably, this process was delayed by tens of millions of years in around a quarter of the genome, which has had a pervasive impact on lineage‐specific genome evolution and adaptive potential (Robertson *et al*. 2017).

A significant percentage of the genome (10–20%) in salmonids has yet to complete the rediploidization process and maintains tetraploid genetic characteristics including potential for tetrasomic inheritance (Allendorf *et al*. 2015). Although such regions are long‐recognized (e.g. Allendorf & Thorgaard 1984), their significance is now becoming better appreciated through application of modern genomics in wild populations (e.g. Waples *et al*. 2016). However, the role played by such regions in influencing commercially relevant trait variation remains unknown, because they are preferentially filtered and removed during genomic analysis (e.g. Limborg *et al*. 2016) and remain challenging to incorporate into standard experimental designs. This rediploidization process is also thought to be the primary cause of a major disparity in recombination rate between males and females; males have very limited recombination over large parts of the genome, but with recombination ‘hotspots' near the telomeres, which tend to be regions showing residual tetraploidy (Allendorf *et al*. 2015). Finally, a key outcome of rediploidization is the retention of at least half of all salmonid genes in duplicated pairs from Ss4R (Berthelot *et al*. 2014; Lien *et al*. 2016); for some gene families, the retention rate of Ss4R gene duplicates is 100% (e.g. Garcia de la Serrana & Macqueen 2018). Additionally, one in five salmon genes belongs to a pair of more ancient gene duplicates retained from Ts3R, leading to highly expanded gene families compared to most non‐teleost vertebrates.

Though fascinating, the complexity of salmonid genomes brings challenges, firstly by adding uncertainty to the quality of reference genome sequences in regions where rediploidization was delayed. In such regions, distinguishing duplicated regions during bioinformatic sequence assembly remains challenging, particularly when using short‐read data (see the later sub‐section ‘Improvements in genome assemblies'). Moreover, the global presence of duplicated regions can reduce confidence when mapping short‐read sequence data to reference genomes, with potential impacts on RNASeq, SNP calling and population genetic analyses (see the section ‘Growing toolbox for genome‐wide investigations'). Interpreting functional signals, especially gene expression, in the face of gene family expansions is likewise challenging, as salmon often retain multiple co‐orthologues of single genes found in model taxa like zebrafish or human. Such duplicated copies are often differentially expressed (e.g. Lien *et al*. 2016; Robertson *et al*. 2017) and can have divergent protein sequences, making it important to interpret their functions and expression as a ‘sum of parts' when establishing the roles of candidate genetic systems under investigation.

## Domestication and selective breeding

Commercial‐scale salmon farming began in Norway in the 1960s, expedited by trials in the early 1970s that demonstrated the huge potential of family‐based breeding programmes (Gjedrem 2012). In these trials, gametes from salmon taken from approximately 40 Norwegian rivers were collected and formed the basis of robust estimations of genetic parameters and the first commercial breeding programme (Gjøen & Bentsen 1997). Other similar breeding programme initiatives were instigated, including the establishment of the Mowi, Rauma, Jakta and Bolaks strains in Norway (Glover *et al*. 2017). Together, following various crossing and international export events, these strains underpin the vast majority of global salmon aquaculture. The consolidation of breeding companies over recent years has resulted in very few but large international players that supply eggs to all the major salmon‐producing countries. These include AquaGen (Norway), Benchmark (UK; owners of both SalmoBreed and StofnFiskr), Hendrix Genetics (Netherlands; owners of Landcatch) and AquaInnovo (Chile), with further consolidation underway via a joint venture between Benchmark and AquaInnovo.

The Norwegian family‐based breeding programmes successfully focussed on increasing growth rate, with estimates of genetic gain per generation of approximately 15% (Gjedrem & Rye 2016). This is vastly superior to terrestrial livestock, albeit the generation interval of salmon is relatively long, typically 3–4 years. This high level of genetic gain may be due in part to the selection intensity associated with the high fecundity of salmon (several thousand offspring per female) and in part to a very recent domestication history, providing high levels of genetic variability influencing traits of importance for farming. In contrast, terrestrial livestock species have been domesticated and selected (directly or indirectly) for favourable traits for approximately 10 000 years (Mignon‐Grasteau *et al*. 2005).

Subsequently, from the 1990s onwards, as breeding programmes became more advanced and needs of producers changed, the breeding goals broadened to include traits such as disease resistance, rate of sexual maturation and fillet characteristics (Gjedrem & Rye 2016). The typical structure of a breeding programme developed to take advantage of the amenable features of salmon biology, in particular external fertilisation and high fecundity. As a result, it was possible to maintain breeding nuclei of approximately 100–300 families, retaining a proportion of juveniles from each family within the nucleus while setting aside their full siblings for production and performance testing. This process is known as ‘sib testing' (short for sibling testing) and enables recording of traits impossible or impractical to measure directly on selection candidates in the nucleus (e.g. resistance to specific pathogens or invasive fillet traits). In addition, technology advances began enabling genetic markers to be applied to capitalise on the within‐family component of genetic variation in addition to the between‐family component. The first example of this was the extensive use of marker‐assisted selection for favourable alleles at a major QTL explaining the vast majority of variation in host resistance to infectious pancreatic necrosis virus (IPNV) (Houston *et al*. 2008, 2010; Moen *et al*. 2009; Gheyas *et al*. 2010). The result was a sustained decrease in the incidence of IPN outbreaks to near zero and widespread recognition of the potential of (molecular) genetics in selective breeding to tackle infectious disease (Norris 2017). Subsequent studies have demonstrated that most other traits of importance for salmon production are heritable but highly polygenic (for reviews, see Yáñez *et al*. 2014; Houston 2017), and therefore genomic selection (GS) is considered the state of the art for application of genomics to genetic improvement (see the sub‐section ‘Mapping QTL and genomic selection').

Due to the outcomes of domestication and selective breeding, there are both genetic and phenotypic differences between wild and farmed salmon populations. Escapees from salmon farms are thought to have resulted in significant introgression into wild stocks, which may impact life‐history traits and the subsequent fitness of natural populations (e.g. Glover *et al*. 2017). As such, approaches to prevent interbreeding of wild and farmed fish are being developed, including mass generation of triploids (Benfey 2001) and gene editing to induce sterility in farmed stocks (see the sub‐section ‘Genome editing for understanding and improving traits'). Comparisons of farmed and wild stocks are useful for detecting genetic signatures of domestication. Salmon present an interesting model due to the passage of relatively few generations since organised farming began, perhaps around 13 generations. Comparisons between the genomes of farmed and wild populations have revealed selection signals related to various domestication‐related traits, affecting genes associated with growth, early sexual maturation and immune response (Gutierrez *et al*. 2016; Liu *et al*. 2017b).

## Growing toolbox for genome‐wide investigations

High‐throughput sequencing has transformed salmon genetics, in particular the ease of generating genome‐wide genetic marker datasets. A major step forward came with the arrival of restriction site‐associated DNA sequencing (RAD‐seq; Baird *et al*. 2008) and subsequent variations. The cost‐effective discovery and concurrent genotyping of multiple, multiplexed samples in a single Illumina sequencing lane has been widely applied in many salmonid species (reviewed by Robledo *et al*. 2017, 2018a). RAD‐seq and similar genotyping‐by‐sequencing techniques were applied in salmon even before the availability of a reference genome and have been used for QTL mapping, linkage mapping, genome‐wide association (GWA) studies, population genetics and SNP discovery for creating genotyping tools, including SNP arrays (Robledo *et al*. 2017, 2018a). Subsequently, high‐density SNP arrays were published for salmon (Houston *et al*. 2014; Yáñez *et al*. 2016), in addition to multiple unpublished custom arrays used in research and development projects by individual breeding companies. These arrays have enabled many high‐resolution genetic association and population genetic studies (e.g. see the next section ‘Modern applications of genomics'), in addition to the first tests of GS in salmon breeding programmes (Ødegård *et al*. 2014; Tsai *et al*. 2015b). Whole‐genome (re)sequencing (WGS) methods have also been applied for variant detection and calling in salmon but remain expensive, and population‐scale genotyping by WGS requires further research (see the sub‐section ‘Moving towards WGS for population analysis'). Genetic marker resources have been utilized to develop linkage maps of the salmon genome, including high‐density SNP linkage maps created using SNP arrays (Lien *et al*. 2011, 2016; Tsai *et al*. 2016a) and RAD‐seq (Gonen *et al*. 2014).

The landmark publication of the salmon genome (Lien *et al*. 2016) provided a reference assembly that advanced possibilities for high‐resolution genomic analyses. Genome‐wide gene expression profiling has traditionally been performed in salmon by microarrays, and these reliable tools are still widely applied (e.g. Król *et al*. 2016; Robledo *et al*. 2016; Vera *et al*. 2017). However, RNASeq performed against the reference genome is now routinely used for functional genomic investigations focussed on evolution (e.g. He *et al*. 2017; Robertson *et al*. 2017), aquaculture (e.g. Robledo *et al*. 2017) and physiology (e.g. Gillard *et al*. 2018). Mapping against a reference genome, compared to a transcriptome assembly, also has the benefit that highly similar duplicated regions can be distinguished in the analysis, assuming such regions have been correctly assembled. Conversely, the assembly of transcript sequence data in species with recent WGD is prone to the collapse of contigs and generation of chimeric contigs (e.g. Krasileva *et al*. 2013), which makes RNAseq analyses and interpretation more challenging. Given the wide range of approaches available for RNAseq and other mapping‐based genomic analyses, the field would benefit from a move towards standardizing pipelines and converging on best‐practices to increase comparability across studies. This is one of the goals of the recently established ‘Functional Annotation of All Salmonid Genomes' (FAASG) initiative (Macqueen *et al*. 2017), described fully in the sub‐section ‘Improved annotation and understanding of genome function and regulation'. As increasing quantities of genetic and functional genomic data are generated, a portal for interrogating and visualising these data is necessary for widespread community uptake beyond the standard public repositories, and the genome browser Salmobase.org (Samy *et al*. 2017) is currently serving this purpose.

High‐quality annotations of protein products across a genome also enable investigations applying high‐throughput proteomic approaches that couple liquid chromatography with mass spectrometry to identify huge numbers of putative peptides; data that can be used for quantitative comparisons of protein abundance and modifications (e.g. Breker & Schuldiner 2014). This represents a powerful new tool in the functional genomics armoury for salmonids and is consequently being up‐taken rapidly for investigations of fish physiology and health (e.g. Liu *et al*. 2017a; Causey *et al*. 2018a,2018b; Kumar *et al*. 2018; Nuez‐Ortín *et al*. 2018).

## Modern applications of genomics: sequence to consequence

### Mapping QTL and genomic selection

The genomic toolbox developed for salmon has enabled a wide range of applications in aquaculture breeding and genetics. The case of IPN resistance is one of the most famous examples of a major QTL impacting an economically important trait in a farmed animal species (Houston *et al*. 2008, 2010; Moen *et al*. 2009; Gheyas *et al*. 2010). High‐throughput sequencing approaches have subsequently allowed development of SNP‐based genetic tests to predict IPN resistance of salmon without the need for regular disease challenge experiments (Houston *et al*. 2012; Moen *et al*. 2015). Furthermore, functional studies have been undertaken to highlight the marked differences in gene expression response to infection between resistant and susceptible salmon fry (Robledo *et al*. 2016) and to suggest that epithelial cadherin may be part of the mechanism underlying the QTL (Moen *et al*. 2015). However, in subsequent QTL scans, including GWA studies, there has been little evidence for additional major QTL affecting disease resistance or any other economically important trait (except for maturation; described in the next sub‐section ‘Population genetics to discover the basis of life‐history traits'). For example, significant QTL of relatively minor effect have been identified for salinity tolerance (Norman *et al*. 2012), body weight (Houston *et al*. 2009; Gutierrez *et al*. 2012, Tsai *et al*. 2015a; Yoshida *et al*. 2017) and resistance to several diseases and pathogens, namely pancreatic disease (Gonen *et al*. 2015), salmon rickettsial syndrome (Correa *et al*. 2015), amoebic gill disease (Robledo *et al*. 2018b) and sea lice (Correa *et al*. 2016; Tsai *et al*. 2016b). The percentage of genetic variation (heritability) explained by the identified QTL in all these studies was low (between 2 and 20%, compared to 80–100% for IPN resistance), and therefore marker‐assisted selection is unlikely to be a fruitful strategy for improving these target traits.

Genomic selection was first described by Meuwissen *et al*. (2001) and involves the use of genome‐wide genetic marker data to predict breeding values for selection candidates. The premise of GS is that marker effects are estimated in a ‘training' population that has been measured for both phenotypes and genotypes, and the model developed is used to predict breeding values for individuals with genotype information only. GS has transformed the livestock breeding industry, generating substantially faster genetic gain for key economic traits compared to the traditional pedigree‐based approach (Meuwissen *et al*. 2013). Applications of GS in aquaculture began with the development of the first high‐density SNP arrays, containing hundreds of thousands of SNPs (Houston *et al*. 2014; Ødegård *et al*. 2014; Yáñez *et al*. 2016). The focus of GS in salmon has been on disease resistance due to its economic importance and the practical impossibility of trait measurement on the selection candidates themselves. In all published GS studies in salmon, the results have shown higher prediction accuracy of breeding values than with pedigree information alone (Ødegård *et al*. 2014; Tsai *et al*. 2015b, 2016a,2016b; Bangera *et al*. 2017; Robledo *et al*. 2018b). A major downside to GS is that high‐density genotyping in large numbers of individuals can be prohibitively expensive. Approaches to reduce genotyping costs, such as the use of low‐density marker panels, including with genotype imputation, have shown promising results (Tsai *et al*. 2017; Yoshida *et al*. 2018). GS has been shown to be effective in salmon for which the training and test populations are closely related (such as in a typical sib‐testing scheme), but the ability to predict breeding values in animals more distantly related to the training population is rather limited (Tsai *et al*. 2016b) and may require new approaches including increased focus on potential functional variants, such as those identified under FAASG initiative studies (see the sub‐section ‘Improved annotation and understanding of genome function and regulation').

### Population genetics to discover the basis of life‐history traits

The latest genomic tools have also been used to reveal the genomic basis of salmon traits with significance for adaptation in natural environments. As a prime example, which also has significance for aquaculture, two closely timed publications identified a major locus (harbouring the *vgll3* gene) explaining a large proportion (approximately one third) of individual variation in the age that salmon undergo maturation, which is under divergent selection in males and females. Both investigations used GWA methods to locate the same genomic region, either using WGS following pooling of individuals from multiple populations (Ayllon *et al*. 2015) or by applying a high‐density SNP array on a large number of populations and subsequent WGS to interrogate potential functional variants (Barson *et al*. 2015). The latter study provides a classic example of how sexual conflict—when selection acts in different directions in males and females—can be partly resolved by balancing selection on a single autosomal gene (Mank 2017). Follow‐up studies are providing insights into the mechanisms by which *vgll3* is operating in reproductive systems, providing evidence for distinct regulation between sexes (Kjærner‐Semb *et al*. 2018). Another recent study applied WGS using pooling of individuals to identify highly differentiated regions of the genome that harboured genes with important immune functions, comparing Northern and Southern populations of salmon in Norway (Kjærner‐Semb *et al*. 2016). Similarly, the same high‐density SNP array applied by Barson *et al*. (2015) was recently applied to identify candidate genomic regions and genes under divergent selective pressures in sub‐populations of salmon inhabiting the Teno River in Finland (Pritchard *et al*. 2018).

## State of the art and perspectives

### Improvements in genome assemblies

As reviewed in the section ‘Growing toolbox for genome‐wide investigations', it is an exciting time for genome‐enabled biology in salmon. This sentiment extends to other Salmonidae members, for which high‐quality and draft genomes have been published, including for rainbow trout (*Oncorhynchus mykiss*; Berthelot *et al*. 2014), Chinook salmon (*Oncorhynchus tshawytscha*; Christensen *et al*. 2018a; Narum *et al*. 2018), European grayling (*Thymallus thymallus*; Varadharajan *et al*. 2018) and Arctic charr (*Salvelinus alpinus*; Christensen *et al*. 2018b), or are available yet currently unpublished, including for coho salmon (*Oncorhynchus kisutch*; NCBI accession no. GCA_002021735) and Danube salmon (*Hucho hucho*; NCBI accession no. GCA_003317085). Several of these assemblies have been anchored to chromosomes and are annotated to a high standard (Macqueen *et al*. 2017). Such resources provide a powerful framework that, when coupled with our understanding of phylogeny (e.g. Macqueen & Johnston 2014; Lecaudey *et al*. 2018), will enable salmon researchers to harness comparative approaches to reconstruct the evolutionary origins of traits of commercial and evolutionary relevance—interesting in the context of Ss4R and the diversity of ecological adaptations present among salmonid lineages (Robertson *et al*. 2017; Varadharajan *et al*. 2018). However, despite such substantial recent progress, improvements to existing genome assemblies and annotations will be vital to more fully exploit genomic information in salmon and related species.

Central to improvement of genome assemblies are technological advancements, which salmonid researchers have been quick to capitalise on, for example by incorporating long‐read data generated on Pacific Biosciences (PACBIO) platforms (e.g. Lien *et al*. 2016; Christensen *et al*. 2018a,2018b). This trend will continue, ensuring improvement in the annotation of poorly represented regions in salmon genomes, notably repetitive and tetrasomic regions. Although PACBIO and some classes of short‐read data (e.g. mate‐pair libraries) provide long‐range information that facilitates resolution of complex regions in genome assemblies, emerging approaches hold greater advantages in the same respect. Nanopore sequencing on Oxford Nanopore Technologies (ONT) platforms, including the portable MinION sequencer, generates ultra‐long reads that reach lengths beyond PACBIO's capabilities and is being successfully applied to assemble large and complex eukaryotic genomes (e.g. Jain *et al*. 2018; Michael *et al*. 2018). The ONT approach is currently being developed by several salmon research groups and is considered to hold great promise going forward.

Additional tools that provide the long‐range information necessary to improve reference genome assemblies include high‐throughput chromatin conformation capture (Hi‐C), which generates genome‐wide data on chromatin interactions that can be applied to scaffold existing assemblies to a high level (e.g. Burton *et al*. 2013; Putnam *et al*. 2016), an approach applied in an improved assembly of the rainbow trout genome (accession no. GCA_002163495; unpublished). Optical mapping similarly generates very long‐range genomic information that can be used to improve complex genome assemblies (reviewed by Howe & Wood, 2015), though as far as we are understand, is yet to be applied in salmonids in published studies. A highly promising tool for salmon research is ‘linked‐read' sequencing, using the 10× genomics microfluidic platform to partition fragmented genomic DNA into large molecules that are subsequently sequenced as short reads that retain a unique barcode matching the original fragment (Zheng *et al*. 2016). This approach can be used to generate assemblies that distinguish both chromosome sets (i.e. a ‘diploid assembly') (Weisenfeld *et al*. 2017). Generation of such long‐range haplotype information would have major applications in salmon population genomics and in theory could be used to distinguish tetrasomic regions directly during sequence assembly. It is also crucial to note that the merging of data gathered across the range of established and emerging sequencing platforms is essential for fully exploiting the unique advantages of different approaches while offsetting their varying limitations (e.g. using highly accurate short‐read data to clean up ultra‐long sequence data that currently suffer from high error rates).

### Improved annotation and understanding of genome function and regulation

A further step advance in understanding of how variation in the blueprint of the salmon genome leads to trait variation will require improved knowledge of genome function and the complex regulation of gene expression. Following in the footsteps of the FAANG initiative for terrestrial farmed animals (Andersson *et al*. 2015), the FAASG initiative was established to improve knowledge of genome function for salmonids (Macqueen *et al*. 2017). FAASG is a community‐led initiative that will harness modern experimental molecular biology and sequencing technologies to identify and characterise functional elements in the genome. This will include studies of polymorphic variation within species, fixed variation across species, gene expression phenotypes covering multiple RNA classes and their variants, epigenetics and gene expression regulation, along with protein‐level variation.

The epigenetic molecular component of phenotypic variability in salmonids is relatively poorly understood but holds promise for translational research relevant to stock enhancement in aquaculture (for recent reviews, see Gavery & Roberts 2017; Best *et al*. 2018). In this respect, FAASG aims to exploit a range of well‐established technologies that enable profiling of DNA methylation, repressive and permissive histone modifications, chromatin accessibility and higher chromatin structure (Macqueen *et al*. 2017). The salmonid research community is already applying several of these approaches. For example, a recent study integrated transcriptomics with genome‐wide epigenetic analyses to demonstrate remodelling of methylation status due to stress (Moghadam *et al*. 2017). A role for global changes in methylation in shaping phenotypic variation in response to the environment was also proposed recently with respect to the reduced fitness observed in hatchery‐reared salmon used to re‐stock wild populations (Le Luyer *et al*. 2017). Moreover, the possible role of histone modifications for the thermal dependence of salmon immune responses was recently reported (Boltana *et al*. 2018). In addition, an increased understanding of variation in the salmon microbiome will be important for improving our understanding of its role in complex traits, and this is of increasing interest for salmon biologists (e.g. Gajardo *et al*. 2016; Dehler *et al*. 2017; Uren Webster *et al*. 2018). Microbiome composition is almost certain to influence genome functional and epigenetic responses, with resulting impacts on phenotype, but there remains much left for fish biologists to learn in this area, with many promising avenues for genomic investigations (e.g. Llewellyn *et al*. 2014; Ghanbari *et al*. 2015).

A unique feature of the FAASG initiative is that functional annotation will facilitate an improved understanding of genome functional evolution after the Ss4R event. At the population scale, an improved understanding of genome function will allow prioritisation of polymorphisms that may be expected to have direct effects on traits of interest, rather than simply as genetic markers. Further, it will enable shortlisting of candidate variants for use with gene‐editing technologies to demonstrate function and potentially improve traits for aquaculture (see the sub‐section ‘Genome editing for understanding and improving traits').

### Moving towards WGS for population analysis

Population‐scale WGS has the potential to significantly enhance understanding of the genetic basis of traits of evolutionary and economic interest. Although genotyping‐by‐sequencing techniques, such as RAD‐seq, have been widely applied (Andrews *et al*. 2016; Robledo *et al*. 2017), the ongoing reduction in sequencing and high‐power computing costs is expected to make WGS routine in the future. Studies using pooled WGS have been applied to investigate signatures of selection (Kjærner‐Semb *et al*. 2016) and to map a major QTL affecting maturation (Ayllon *et al*. 2015). However, individual‐level population‐scale WGS can offer insights including the role of different types of polymorphic variation in trait architecture (e.g. structural variants including copy number variation, inversions, etc.) and would enable the study of rare and *de novo* variants that are unlikely to be detected using SNP arrays due to ascertainment bias. To be affordable in the short term for population datasets, WGS can be performed at low individual coverage. This raises issues with potentially erroneous variant calling due to sequence errors and/or heterozygous sites being called as homozygous due to sequencing of just one allele (Bilton *et al*. 2018). Harnessing pedigree information together with imputation approaches within a breeding programme may be an effective route for improving the quality of low‐coverage WGS data and may have downstream benefits for genomic prediction accuracy (Hickey 2013). When combined with GWA approaches and the functional annotation described above, WGS can provide the means to discover and characterise candidate causative variants within QTL regions that can be selected for functional testing.

### Genome editing for understanding and improving traits

Genome editing technologies allow targeted changes to the genomic DNA at a specific location, and engineered CRISPR/Cas9 systems (Cong *et al*. 2013; Mali *et al*. 2013) are widely applied for this purpose. The Cas9 enzyme makes a double‐stranded cut at a specific target site enabled by the guide RNAs. The resulting DNA changes are the result of two major categories of DNA repair mechanisms. The first of these is non‐homologous end joining (NHEJ), whereby the repair mechanism does not require a homologous template and will result in small insertions or deletions at the cut site that can result in loss‐of‐function mutations. The second is homology‐directed repair (HDR), whereby a DNA template is provided that is similar to the flanking sequence of the cut site (but may contain a user‐targeted change in sequence), and the cell uses the template to repair at the cut. The successful use of CRISPR/Cas9 with NHEJ to generate *slc45a2* knockout salmon in the F0 generation via microinjection into one‐cell stage embryos demonstrated the efficacy of the technology in salmon (Edvardsen *et al*. 2014). Subsequent studies have successfully applied CRISPR/Cas9 to generate sterile salmon via ablation of germ cells caused by *dnd* knockout (Wargelius *et al*. 2016). In addition to these *in vivo* successes, CRISPR/Cas9 has been successfully applied for gene knockout in a salmonid cell line (CHSE‐214, Dehler *et al*. 2016). Evidence for targeted changes made via incorporation of a template DNA using HDR has not yet been published for salmon, though such work is currently underway in several groups. ‘Base editing' is another emerging gene editing approach that can make specific targeted changes in the genome without the need to cut the genomic DNA or utilize a template DNA (Komor *et al*. 2016) and has been successfully applied in zebrafish (Zhang *et al*. 2017) but is yet to be trialled in salmon as far as we are aware.

There are a number of potential future applications of genome editing for increasing understanding of salmon biology and improving traits of importance for salmon production and welfare. Genome‐wide screening approaches, including the use of the genome‐scale CRISPR knockout (GeCKO) technique (Shalem *et al*. 2014), may facilitate identification of genes involved in traits of importance, particularly traits that can be measured in cell cultures (e.g. resistance to viral disease). GeCKO involves lentiviral delivery of a library of tens of thousands of unique guide RNAs into cell cultures for genome‐wide gene knockout followed by negative or positive selection screening (Shalem *et al*. 2014). There are technical hurdles to overcome before GeCKO screens could be applied in salmon, in particular relating to delivery of guide RNAs, as lentiviruses are not considered an effective delivery method in salmon cells. CRISPR/Cas9 is also likely to be used to test hypotheses relating to causative variants underlying QTL. Ideally, HDR or base‐editing approaches could be applied to ‘swap' one version of the allele at the candidate variant for the alternate version before assessing the impact on the trait of interest. For all editing approaches, it is important to consider, and if possible exclude, potential off‐target effects, which remains a contentious issue in medical research (Nutter *et al*. 2018). However, there are several exciting potential applications of genome editing in salmon breeding programmes (subject to public and regulatory acceptance; see the following sub‐section) which could include (i) fixing of favourable alleles at QTL affecting traits of economic interest; (ii) rapid ‘introgression' of favourable alleles from other populations, strains or species into a salmon breeding population; and (iii) creation of ‘*de novo'* alleles based on knowledge of the biology of the trait in question. For the latter application, an example from terrestrial livestock is the removal of an exon of the *CD163* gene in pigs, which results in complete resistance to the porcine reproductive and respiratory syndrome virus (PRRSV) (Burkard *et al*. 2017).

### Regulatory and public perception landscape

Finally, it is important to briefly consider on‐going changes surrounding the regulation and uptake of genetically modified (GM) or gene‐edited (GE) salmon for production in aquaculture. These methods have the potential to rapidly introduce favourable traits (as described above) and to provide solutions to major challenges faced by the salmon aquaculture sector. However, there clearly are regulatory and perception issues to consider, and these include the definition of what constitutes GM and the extent to which gene editing should be considered separately and/or split into different categories according to the nature of the induced change to the genome. These decisions will need to involve a wide variety of stakeholders, including in the aquaculture and retail industries, policymakers, consumers and other members of the public. At one end of the scale, it is now possible to generate GE animals with single base changes in the genome that are already segregating in wild populations. At the other end of the scale are more radical changes in the genome that are absent (or perhaps rare) in nature (e.g. the PRRSV example in pigs). Clearly, there are many scenarios in between, and the challenge is to find a balance that allows the revolutionary potential of gene editing to be realized in an objective (i.e. scientifically informed) manner with appropriate regulatory frameworks. Although arguments have been presented that gene editing for alleles that occur naturally in agricultural populations should not be considered gene modification even under strict legal frameworks (Custers 2017), the recent ruling by the European Court of Justice that GE crops should be considered GM organisms is a major setback (Callaway 2018). However, a landmark was set recently by the approval of a GM salmon strain as fit for human consumption by the US Food and Drug Administration and the Canadian Food Inspection Agency (and for sale by the latter) after a long period of regulatory limbo (Waltz 2017). The AquaAdvantage (AquaBounty Technologies) strain shows enhanced growth due to the integration of a growth hormone (GH) gene from Chinook salmon linked to a promoter from another fish species that drives high GH expression. Ultimately, research and development relating to potential uses of gene modification and gene editing in aquaculture will continue to develop rapidly and should do so in parallel with an extensive dialogue between the various stakeholder groups described above to help establish a knowledge‐driven regulatory framework for future applications.
